# Normal mammary epithelial cells promote carcinoma basement membrane invasion by inducing microtubule-rich protrusions

**DOI:** 10.18632/oncotarget.4728

**Published:** 2015-08-03

**Authors:** Meng-Horng Lee, Pei-Hsun Wu, Daniele Gilkes, Ivie Aifuwa, Denis Wirtz

**Affiliations:** ^1^ Johns Hopkins Physical Sciences - Oncology Center, The Johns Hopkins University, Baltimore, Maryland 21218, USA; ^2^ Department of Chemical and Biomolecular Engineering, The Johns Hopkins University, Baltimore, Maryland 21218, USA; ^3^ Departments of Pathology and Oncology and Sidney Kimmel Comprehensive Cancer Center, The Johns Hopkins School of Medicine, Baltimore, Maryland 21205, USA

**Keywords:** cancer invasion, cell-cell interaction, protrusion, microtubule, laminin

## Abstract

Recent work suggests that the dissemination of tumor cells may occur in parallel with, and even preceed, tumor growth. The mechanism for this early invasion is largely unknown. Here, we find that mammary epithelial cells (MECs) induce neighboring breast carcinoma cells (BCCs) to cross the basement membrane by secreting soluble laminin. Laminin continuously produced by MECs induce long membrane cellular protrusions in BCCs that promote their contractility and invasion into the surrounding matrix. These protrusions depend on microtubule bundles assembled *de novo* through laminin-integrin β1 signaling. These results describe how non-cancerous MECs can actively participate in the invasive process of BCCs.

## INTRODUCTION

Local invasion is the first and critical step in cancer metastasis. Carcinoma cells must breach the basement membrane barriers in order to invade lymphatic or blood vessels located within the interstitial stroma, which mediates their distant metastasis [1]. Accumulating clinical observations suggest that the formation of long cellular protrusions is critical for cancer invasion [2, 3]. Several recent *ex vivo* studies recapitulate this protrusive morphology of cells in three dimensional (3D) stromal type I collagen [4–9]. In contrast, most cells that fail to form protrusions demonstrate inefficient invasion in gels comprised of basement membrane proteins [10–12]. Here, we hypothesize that specific soluble factors may be required to sufficiently stimulate carcinoma cells to form invasive protrusions to overcome the basement membrane barrier.

Accumulating evidence suggests that normal cells actively participate in tumorigenesis and influence cancer invasion [13–16]. For example, immune cells [17] (e.g. macrophages) and cells of the connective tissue (e.g. cancer-associated fibroblasts) are coopted and recruited by tumor cells to enhance the growth and invasion of cancer cells [18]. However, recent work suggests that the dissemination of individual cancer cells may occur in parallel with tumor growth [19–21], and not following tumor growth. The mechanism by which cancer cell invasion precedes tumor growth is a matter of intense investigation. Upon oncogenic transformation and before invasion, a large fraction of carcinoma cells are in contact with non-cancerous epithelial cells [22, 23]. We previously demonstrated that mammary epithelial cells (MECs) surrounding a single carcinoma cell promote the spreading of that cell within the normal epithelium [24]. Here, we asked whether surrounding MECs also participated in the early invasion of breast carcinoma cells to cross the basement membrane, the first barrier that separates cancer cells surrounded by normal epithelial cells and connective tissues.

Our results indicate that MECs actively prompt neighboring BCCs to cross the basement membrane. Soluble factors secreted by MECs induce the formation of long thick membrane protrusions that endow BCCs with an aggressively invasive phenotype. Biochemical analysis suggests that soluble laminins secreted by MECs is responsible for this invasive, protruded phenotype. Importantly, MECs are not coopted by the cancer cells to produce laminins. Rather, MECs continuously secrete soluble laminins, which does not influence their own physiology. MEC-induced protrusions in BCCs are filled with thick microtubule bundles wraped by a thin cortex of actin filaments, a cytoskeletal organization distinct from that in standard cellular protrusions, including filopodia and lamellipodia. The induction of long microtubule-rich extensions is required for the invasion of BCCs across the basement membrane. These results suggest that the migration of BCCs through the basement membrane and early local invasion depend critically on biochemical stimulation from neighboring MECs.

## RESULTS

### Non-cancer epithelial cells induce an invasive phenotype in carcinoma cells

**Figure 1:**
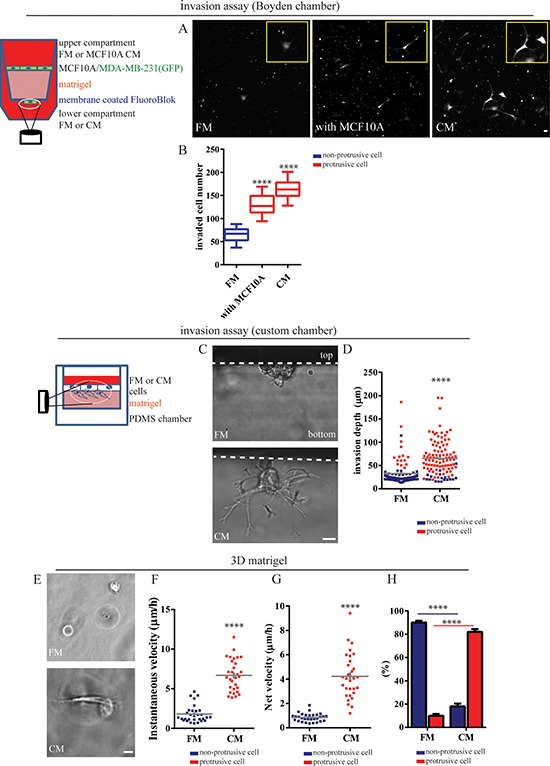
Mammary epithelial cells induce an invasive phenotype in breast carcinoma cells **A.** and **B.** MDA-MB-231cells stably expressing EGFP were seeded alone or with MCF10A cells on top of a Boyden chamber coated with a thick layer of matrigel. During invasion, we added the same (regular fresh medium, FM or MCF10A conditioned medium, CM) amount of medium in the upper and lower compartments of the chamber. (A) Representative images of invaded MDA-MB-231 cells at the bottom membrane of the chamber. Scale bar, 20 μm. (B) Average number of invaded cells per field of view. **C.** and **D.** MDA-MB-231 cells were placed on the top of matrigel within a custom chamber. (C) Representative morphology of invading cells in FM and CM imaged from the side of the matrix for improved spatial resolution (Scale bar, 20 μm) and (D) invasion depth of individual BCCs in FM or CM. Color coding of the dots specify whether cells were round or protrusive. **E–H.** MDA-MB-231 cells were embedded inside a three-dimensional (3D) matrigel matrix in the presence of FM or CM. (E) Representative spreading morphology of MDA-MB-231 cells in FM (top) or CM (bottom) under phase-contrast microscopy. Scale bar, 20 μm. (F) Fractions of non-protrusive (blue) and protrusive (red) MDA-MB-231 cells inside a 3D matrigel in FM and CM. (G) Instantaneous and (H) net velocity of MDA-MB-231 cells inside the three-dimensional (matrigel) matrix in the presence in FM or CM. ****:*P* < 0.0001.

To investigate whether mammary epithelial cells (MECs) surrounding transformed breast carcinoma cells (BCCs) could actively participate in the invasive process of these BCCs, we devised a co-culture system composed of a single layer of human MECs (MCF10A cells) containing inter-dispersed human BCCs (MDA-MB-231). We found that a significantly increased number of MDA-MB-231 cells invaded through the Boyden chamber coated with matrigel when co-cultured with MCF10A cells (Figure 1B) compared to MDA-MB-231 cells in the absence of MCF10A cells.

We next determined whether direct physical contact between MDA-MB-231 cells and MCF10A cells was required or whether soluble factors secreted by MCF10A cells could be sufficient to promote invasion. Conditioned medium (CM) harvested from MCF10A cells alone was sufficient to promote the invasion of MDA-MB-231 cells to the same extent as in the co-culture system described above (Figure 1A and 1B), indicating that this heterotypic intercellular signaling did not require direct physical contact between cancer and non-cancer cells.

To image invasive cells along the (invasive) *z*-axis with high spatial resolution, we built a redundant parallelepipedic chamber (20 × 5 × 10 mm), where two opposite side faces were made of 0.13–0.16 mm thick coverslips, which enabled us to image invading cells from the side of the matrix (Figure 1C). We confirmed that MDA-MB-231 cells treated with MCF10A CM invaded matrigel more efficiently than non-treated cells. Interestingly, we found that, in the absence of CM, the invasive distance of round MDA-MB-231 cells was minimal and the majority of MDA-MB-231 cells that could invade matrigel formed extended protrusions (Figure 1D). In contrast, stimulation by MCF10A CM switched most MDA-MB-231 cells to a protrusive phenotype and the average invasive depth increased two-fold compared to control cells (Figure 1D).

Next, we placed MDA-MB-231 cells inside 3D matrigel (Figure 1E) to carefully examine 3D migratory behavior and cell morphology in 3D gels. We found that MDA-MB-231 cells that had been rendered protrusive by MCF10A CM efficiently moved through the matrix, while non-protrusive cells in control fresh medium rarely moved, at least within a 16 h imaging time (Figure 1F and 1G; Movie S1 and S2). Notably, in the absence of MCF-10A CM, most MDA-MB-231 cells (>90%) adopted a round morphology and only a small fraction of cells (<10%) were able to extend protrusions, in agreement with a recent study [10]. The treatment of MDA-MB-231 cells with MCF10A CM completely flipped this ratio of non-protrusive-to-protrusive MDA-MB-231 cells (Figure 1F).

### Non-cancer epithelial cells induce a prominent protrusive phenotype in carcinoma cells

**Figure 2:**
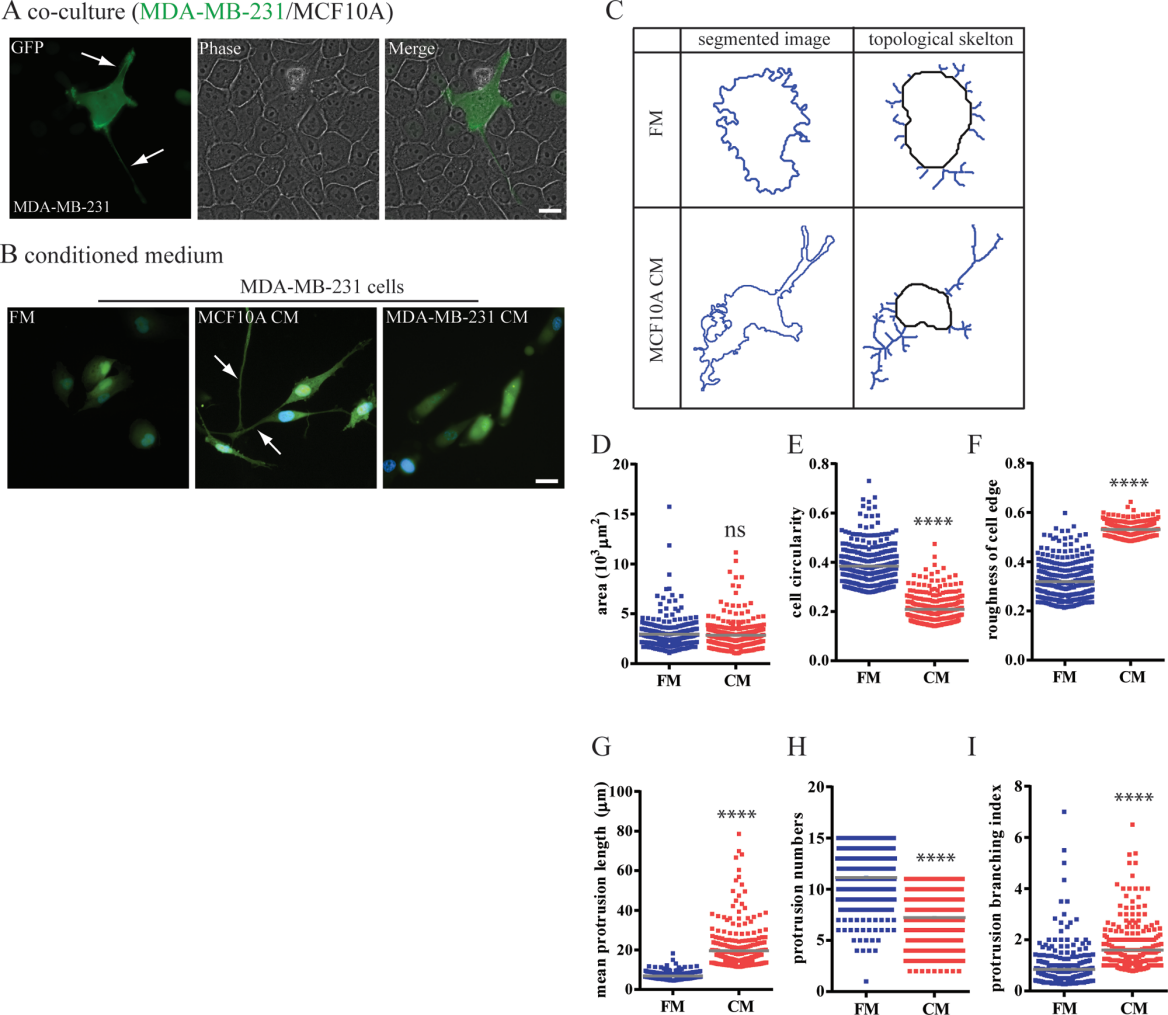
Mammary epithelium cells switch breast carcinoma cells to a highly protrusive phenotype **A.** and **B.** MDA-MB-231 cells stably expressing EGFP were either (A) co-cultured with a layer of confluent MCF10A cells or (B) incubated with MCF10A CM. **C–I.** Computer-segmented MDA-MB-231 cells in FM or CM and associated skeleton plots generated by a custom MATLAB-based software. This skeletonization allows us to unambiguously define the length and number of protrusions per cell. (D) Carcinoma cell spreading area, (E) circularity, and (F) roughness of cell edges in FM and CM were analyzed based on segmented cell images. (G) Mean length of protrusions, (H) mean number of protrusions and (I) protrusion branching index of cells in FM and CM were calculated after conversion of segmented cell images into skeleton plots and automated computation. *n* = 300 cells; ****:*P* < 0.0001.

We asked whether non-cancer mammary epithelial cells (MECs) could actively switch surrounding transformed breast carcinoma cells (BCCs) to a protrusive phenotype. To this end, we co-cultured MDA-MB-231 cells with MCF10A cells. We found that when co-cultured with MCF10A cells, MDA-MB-231cells readily formed long protrusions (Figure 2A) that were largely absent from MDA-MB-231 cells when cultured alone (Figure 2B). Furthermore, conditioned medium (CM) harvested from MCF10A cells was sufficient to induce the protrusive phenotype in MDA-MB-231 cells (Figure 2B, supplementary Movies S3 and S4).

Importantly, this CM did not induce a protrusive phenotype in MCF10A cells themselves (Supplementary Figure S1). Moreover, CM harvested from MDA-MB-231 cells did not induce a protrusive phenotype among themselves or in MCF10A cells (Figure 2B, Supplementary Figure S1A). The same protrusive phenotype in MDA-MB-231 cells was induced by other human MECs, MCF12A (Supplementary Figure S1B–S1D), indicating consistency of our findings. Finally, CM harvested from MCF10A cells induced a protrusive phenotype in BT-549 cells, which are also breast carcinoma cells isolated from the primary tumor (Supplementary Figure S1E–S1G), which again indicated consistency of our findings.

We next determined the extent by which BCCs were made to switch to a protrusive phenotype by MECs. We comprehensively quantified the morphology of MDA-MB-231 cells using automated cell morphometry. Cell spreading area and two additional descriptors of the morphology of cell extensions including cell circularity and roughness of the cell edge were computed. After exposure to CMs from either MCF10A cells or MCF12A cells, 80% of MDA-MB-231 cells demonstrated low circularity (< 0.2) and high roughness of cell shape (> 0.4) (Figure 2D–2F). To consistently define protrusions in an unbiased, unsupervised manner, fluorescent images of cells stained with a non-specific cytoplasmic dye were mathematically converted to topological skeletons, where cell body and protrusions were clearly segmented (Figure 2C) [25]. The average length of protrusions and branching of protrusions both significantly increased in CM-stimulated MDA-MB-231 cells compared to non-stimulated MDA-MB-231 cells (Figure 2G and 2I). In contrast, the number of protrusions slightly decreased in cells cultured in CM (Figure 2H). These results suggest that MDA-MB-231 cells minimize the total number of protrusions to support the formation of few, long protrusions.

### Soluble laminin secreted by non-cancer epithelial cells is required to switch carcinoma cells to a protrusive and invasive phenotype

**Figure 3:**
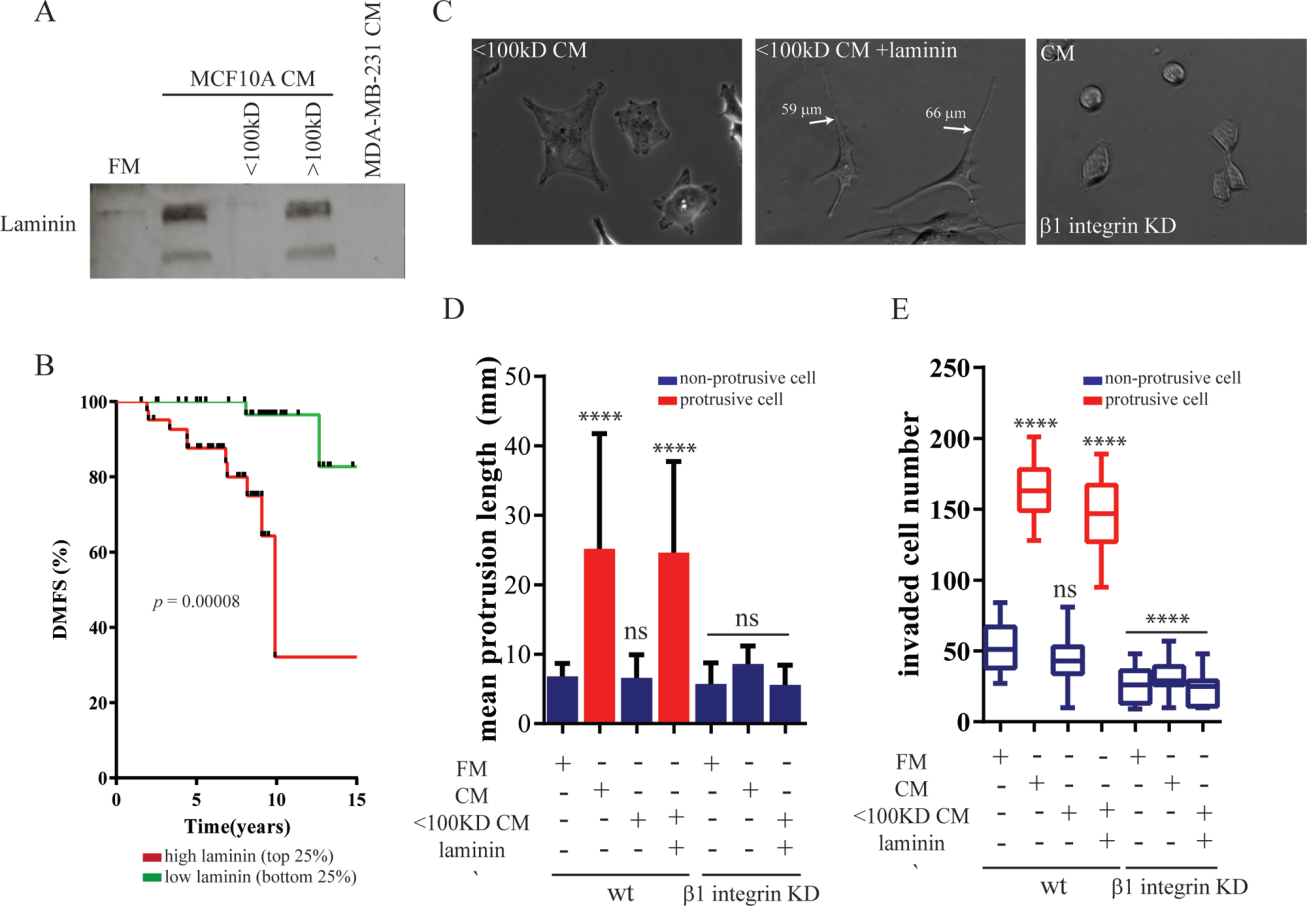
Soluble laminin secreted by mammary epithelial cells induces a protrusive phenotype in breast carcinoma cells **A.** MCF10A-CM was passed through molecular-cutoff filters that selectively excluded molecules above 100 kDa. MCF10A-CM and filtered CM were analyzed by western blotting using an antibody against laminin. **B.** Kaplan–Meier curves were constructed based on the GOBO database to analyze the association of *laminin* mRNA levels with distant metastasis-free survival (DMFS) of breast cancers in the early stage. Statistical analysis was performed using log-rank tests. **C–E.** MDA-MB-231cells were transduced with recombinant lentivirus encoding for shRNA targeting integrin β1 (C) Representative spreading images, (D) mean protrusive length of control or β1 integrin-depleted MDA-MB-231 cells incubated with MCF10A-CM, filtered CM or supplied with 5 μg/ml laminin. (E) Total number of invaded MDA-MB-231 cells in the Boyden chamber coated with a thick layer of matrigel in the same conditions. ****:*P* < 0.0001; **:*P* < 0.01.

To determine the specific factors secreted by MECs that could stimulate BCCs to form long protrusions and invade their surrounding matrix, we passed MCF10A CM through size exclusion filters to segregate molecules with a molecular weight either above 100 kD or above 50 kD in order to estimate the molecular weight range of factors secreted by MCF10A cells. We found that the removal of proteins of molecular weight >100 kD from MCF10A CM prevented the formation of CM-induced protrusions (Figure 3C), as evidenced by a short mean protrusive length in MDA-MB-231 cells (Figure 3D). Correspondingly, the enhanced invasion of MDA-MB-231 was abolished when treated with filtered MCF10A CM (Figure 3E). The results suggested that secreted molecule(s) with a molecular weight >100kD, such as some extracellular matrix (ECM) components, was required to induce the both protrusive and invasive phenotype.

**Figure 4:**
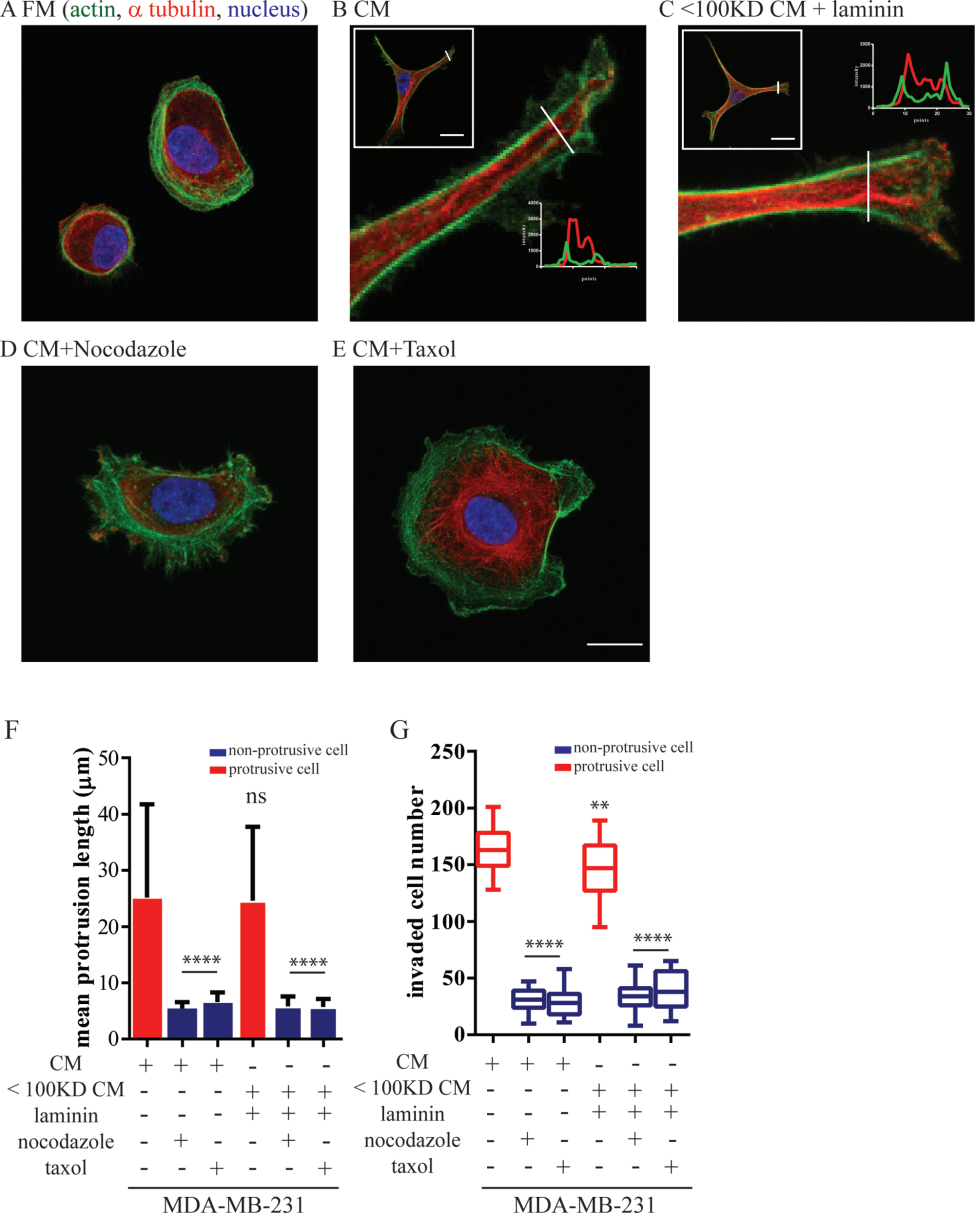
Mammary epithelium cells-induced protrusions depend on microtubule dynamics MDA-MB-231 cells were incubated with MCF10A CM or filtered CM with a cutoff molecular weight of 100 kDA supplied with 5 μg/ml purified laminin in the present or absent of nocodazole (125 nM) and taxol (500 nM). **A–E.** Confocal image of the main protrusion extended from MDA-MB-231. Cells were stained for α-tubulin (red) and F-actin (green); nuclei were visualized with Hoechst 33342 (blue). Scale bar, 20 μm. **F.** Mean protrusive length and **G.** total number of invaded MDA-MB-231 cells through the Boyden chamber coated with a thick layer of matrigel. ****:*P* < 0.0001; **:*P* < 0.01.

Since several studies have indicated a difference in the expression of laminin between MECs and BCCS and have suggestion a function in tumor progression [26–28], we examined whether laminin is involved to promote protrusive and invasive phenotype of BCCs. We confirmed that MCF10A cells secreted a large amount of laminin that was absent from regular fresh medium or MDA-MB-231 CM (Figure 3A). Furthermore, adding purified laminin restored the ability of filtered MCF10A CM depleted of proteins >100 kD to induce a protrusive and invasive phenotype in MDA-MB-231 cells. Meanwhile, the addition of laminin also restored the function of filtered MCF10A CM to promote the invasion of MDA-MB-231 cells (Figure 4E). Notably, the addition of laminin into the regular fresh medium failed to induce long protrusion formation or promote the invasion on MDA-MB-231 cells. These data suggested that laminin present in MCF10A CM was required but not sufficient for the enhanced protrusion formation and invasion of MDA-MB-231 cells.

To further determine the clinical reverence of the correlation between the presence of laminin and early invasion, we examined survival data of breast cancer patients diagnosed with early-stage disease. Patients were stratified according to their expression of laminin. Kaplan-Meier analysis revealed that early-stage breast cancer patients with a high expression of laminin (>75% of median) correlated with decreased distant-free metastasis compared to cancers with low laminin expression (Figure 3B).

Transmembrane cell-receptor integrins α_1_β_1_, α_3_β_1_, α_6_β_1_ and α_6_β_4_ serve as laminin receptors [29, 30]. We found that MDA-MB-231 cells highly expressed mRNA of integrins α_6_ and β_1_ (Supplementary Figure S2A). Therefore, to block laminin-receptor binding we expressed shRNA targeting *Integrin β_1_* or *Integrin α_6_*. Depleting integrin β_1_, but not integrin α_6_, in MDA-MB-231 cells efficiently inhibited the formation of MCF10A (laminin)-induced long protrusions (Figure 4C, and 4D; Supplementary Figure S2B–S2F), suggesting that alpha integrin isoforms are redundancy for laminin binding. Inhibiting MCF10A-induced morphological change by either depleting laminin or abrogating laminin-integrin β_1_ interactions reduced MCF10A-enhanced invasion (Figure 3E). Hence, the formation of protrusions in BCCs by laminin secreted by MECs is required to promote cancer cell invasion.

### The formation of long protrusions in carcinoma cells is essential to promote their invasion

We next determined the molecular mechanism by which BCCs produced long protrusions in the presence of MECs. Confocal optical sectioning showed that the long protrusions in MDA-MB-231 stimulated by MCF10A-CM were mainly composed of microtubule bundles that were circumferentially aligned along the main protrusions. These microtubules were surrounded by cortical actin filaments (Figure 4B and insets) and also aligned along the long axis of the main protrusions. The stimulation of MDA-MB-231 cells supplemented with purified laminin had the same molecular and quantitative effect as CM from MCF-10A cells to promote microtubule-based protrusions (Figure 4C). This cytoskeletal organization is strikingly different from typical lamellipodial structures terminated by small filopodial extensions in control non-stimulated carcinoma cells (Figure 4A) and other types of mesenchymal cells, including fibroblasts and fibrosarcoma cells [31, 32]. This non-conventional cytoskeletal organization in MCF10A-stimulated MDA-MB-231 cells suggested a central role for microtubules in the generation and/or maintenance of their long protrusions.

Inhibition of microtubule formation and dynamics during MDA-MB-231 spreading using the microtubule-depolymerization drug nocodazole and microtubule-stabilizing drug taxol both abrogated protrusion formation, as indicated by the decreased mean protrusive length of MDA-MB-231 cells in the presence of MCF10A cells (Figure 4F). Nocodazole and taxol treatments also eliminated pre-existing, MCF10A-induced long protrusions (Supplementary Figure S3A–S3C), indicating that microtubules supported both the development and the maintenance of long protrusions in MEC-stimulated BCCs. In addition, nocodazole and taxol treatments also reduced MCF10A-enhanced invasion (Figure 4G) in MDA-MB-231 cells, indicating that microtubule-based protrusions are essential for the enhanced invasion of BCCs induced by surrounding epithelial cells.

### The contraction of the carcinoma cell body is necessary for its protrusion-led invasion

**Figure 5:**
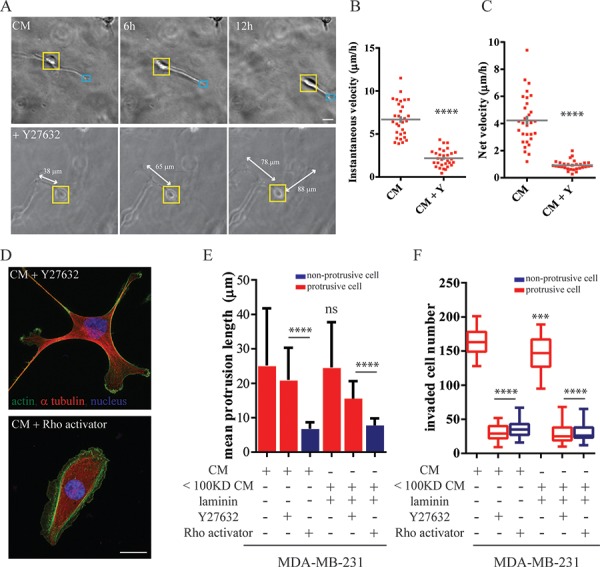
Cell contraction is necessary for protrusion-led invasion **A.** Time-dependent morphological changes of MDA-MB-231 within a 3D matrigel matrix treated with MCF10A-CM or CM supplemented with 10 μM ROCK inhibitor Y-27632. Imaging under phase-contrast microscopy, Scale bar, 20 μm. **B.** Instantaneous and **C.** net velocity of individual MDA-MB-231migrating inside a three-dimensional matrigel matrix. **D–F.** MDA-MB-231 cells treated with MCF10A-CM supplemented with either Y-27632 or Rho activator CN03. (D) Cells were stained for α-tubulin (red) and actin (green). Nuclei were visualized with Hoechst 33342 (blue). Scale bar, 20 μm. (E) The quantitative mean protrusive length and (F) the total number of invaded MDA-MB-231 in the Boyden chamber coated with a layer of thick matrigel. ****:*P* < 0.0001; ***:*P* < 0.001

Next, we asked how the MEC-induced protrusions of BCCs altered migration to maximize their invasion. We found that, when MCF10A-induced protrusive MDA-MB-231cells held their longest protrusion for a long period of time, the cell body still moved or suddenly contracted towards the protrusion tip (Figure 5A and Supplementary Movies S2), which caused the net displacement of the cell. This cell body traction is critical for efficient invasion. Indeed, MCF10A-stimulated MDA-MB-231 cells treated with ROCK inhibitor Y-27632 to inhibit their contractility [11, 33] failed to form an invasive phenotype (Figure 5F and Supplementary Movies S5). Moreover, the addition of a Rho activator CN03 in CM-stimulated MDA-MB-231 cells also prevented cells to invade matrigel (Figure 5F). We found that Rho activation by CN03 in MCF10A-CM prevented MDA-MB-231 cells to form a protrusive morphology and reduced their ability to invade (Figure 5D and 5E). Across all conditions we examined in this study, we found that invasive BCCs were accompanied by a protrusive morphology, indicating that the formation of microtubule-based protrusions is pre-required for MEC-induced invasion of BCCs.

## DISCUSSION

Here, we provide evidence that non-cancer mammary epithelial cells (MECs) can actively enhance the ability of breast carcinoma cells (BCCs) to cross the basement membrane. MEC-induced enhanced invasion is achieved by inducing the formation of long, persistent membrane protrusions in BCCs. This stimulation is unidirectional, going from the normal epithelial cells to cancer cells, i.e. this is not a result of a cooptation by cancer cells. This protrusive phenotype is triggered by laminin-integrin interactions at the surface of carcinoma cells, as evidenced by removing laminin from the conditioned medium obtained from MECs or by depleting the laminin-receptor β1 integrin in carcinoma cells. The protrusive morphology of carcinoma cells is initiated and maintained by microtubule bundles, while the inhibition of actin dynamics causes only a marginal effect on these long protrusions.

Clinical investigations have correlated the presence of laminin to breast cancer invasion [34, 35]. Our results suggest that laminin secreted by MECs is sufficient to promote the invasion of BCCs, arguing that it may not be necessary for these cells to secret their own laminin [36, 37]. In addition, the fact that CM harvested from MECs is sufficient to induce a combined protrusive/invasive phenotype in carcinoma cells further indicates that laminin secreted by epithelial cells does not require paracrine stimulation from carcinoma cells. What determines laminin-mediated invasion is whether carcinoma cells form long, microtubule-filled protrusions in the laminin-rich environment provided by surrounding MECs. Inhibiting morphological changes induced in carcinoma cells by either interfering with laminin-integrin interactions or inhibiting microtubule dynamics prevents laminin-mediated cancer cell invasion.

Interestingly, MEC-stimulated protrusions in carcinoma cells feature a unique cytoskeletal organization, comprised of extended, tightly packed microtubule bundles which form a core surrounded by a dense cortex of actin filaments. Our results highlight several important mediators for these functionally relevant long protrusions. First, laminin-integrin interactions are sufficient for the formation of microtubule bundles. Indeed, BCCs lacking integrin β1 fail to extend microtubules to the cell cortex in laminin-enriched conditioned medium. This finding is further supported by a recent study showing that laminin-integrin interactions promote the localization of microtubule-associated proteins (MAPs) to the cell cortex, which anchor microtubules to the cell edges [38]. Secondly, the deactivation of Rho is necessary for protrusion formation, as evidenced that the addition of Rho activator prevents BCCs to form microtubule-based protrusions in CM. Interestingly, several studies demonstrate that the addition of laminin cause the inactivation of Rho in neuron cells [39–42]. Hence, a full mechanism of microtubule assembly and bundling and Rho signaling may be managed by laminin-integrin β1 interactions.

The commonly accepted dogma considers invasion as a late event in breast tumor progression [20]. However, this conventional wisdom has been challenged by clinical studies showing the existence of early dissemination as evidenced by the appearance of metastasis after surgical removal of small breast tumors in node-negative breast cancer patients and the metastasis of unknown primary tumor [43–45]. The effect of MECs could provide a mechanism allowing the early invasion of BCCs. Particularly, the effect of MECs would efficiently affect individual carcinoma cells, circumventing a requirement for a larger tumor to form and generate sufficient diversity for effective invasion [46].

## MATERIALS AND METHODS

### Cell culture and co-culture

Non-cancerous human breast epithelial cells (MCF10A and MCF12A), transformed metastatic human breast carcinoma cells (MDA-MB-231 and BT-549) were obtained from American Type Culture Collection (ATCC, Manassas, VA). MCF10A and MCF12A were cultured and passed in 5% horse serum supplemented with 20 ng/ml hEGF, 10 μg/ml insulin, 100 ng/ml cholera toxin, and 0.5 μg/ml hydrocortisone. MDA-MB-231 and BT-549 cells were maintained in DMEM supplemented with 10% fetal bovine serum. MCF7 cells were maintained in DMEM supplemented with 10% fetal bovine serum and 10 μg/ml insulin.

The co-culture experiment was performed as previously described [24]. Briefly, MDA-MB-231 cells were co-seeded with MCF10A cells in a 1:50 ratio in MCF10A growth medium. The cell morphology was monitored using fluorescence microscopy following two days of cell culture.

For harvesting conditioned medium (CM), 3 × 10^6^ non-cancerous epithelial cells were seeded in a 10 mm-culture dish. After 2 days culturing, conditioned medium was collected and immediately filtered through a 0.45-μm filter (Millipore, Bedford, MA, USA) to remove cells debris. To maintain consistent conditions, MDA-MB-231 cells seeded with MCF10A medium was used as experimental control. To remove high molecular weight proteins, MCF10A CM was passed through the Amico Ultra centrifugal filter (Millipore).

### Automated quantification of protrusive cell morphology

The cellular morphological features were analyzed from fluorescent images using a custom-MTALAB-based software [47, 48]. First, the boundary of each individual cell stained for F-actin was segmented. The “roughness” of cell shape was computed by recording the positions of points along the cell boundary and then computing the ratio of the standard deviation to the mean of the distance of each point to the cell center. This roughness is low for a cell with a smooth morphology, and high for a cell showing many protrusions. To further quantify protrusive features, cell morphology and protrusions were mathematically converted into topological skeletons [25]. A line that extended from the cell body was defined as a protrusion. The length of each individual protrusion and the total number of protrusions per cell was computed.

### Lentivirus production and transduction

Lentiviral vector expressing shRNA targeting *Integrin β1* was a gift from Dr. Gregory D. Longmore's lab. The shRNA vector targeting *Integrin α6* was purchased from Sigama-Aldrich (St. Louis, MO, USA). The second generation lentiviruse was produced as previously described [49]. Briefly, 293T cells (ATCC) were transiently co-transfected with three plasmids including lentiviral vector, (R 8.91 and pMDG-VSVG using calcium phosphate precipitation method. After 22–24 h transfection, the medium was replaced with fresh medium and lentiviral particles were harvested 24 h later and immediately filtered through 0.45 μm filter (Millipore) to remove cells debris, then stored at −80°C. For transduction, 1 × 10^5^ cells in 35-mm culture dish were repeatedly transduced with lentivirus with 8 μg/ml polybrene to reach high transduction (>80%).

### Immunofluorescence imaging and live-cell microscopy

For immunofluorescence staining, cells were fixed with 4% paraformaldehyde, permeablized with 0.3% triton x-100, and incubated with the mouse anti-α-tubulin (1:5000, room temperature for 1 h, Abcam, Cambridge, MA, USA), phalloidin (1:40, room temperature for 20 min; Invitrogen, Eugene, OR), and Hoechst33342 (1:1000, room temperature for 10 min; Invitrogen). Images of the stained cells were acquired using Nikon A1 confocal microscope (Nikon, Melville, NY, USA) equipped with a 60x plan lens (N.A. 1.2).

For live-cell imaging, cells were embedded in matrigel and plated in a 96-well glass bottom dish (MatTek, Ashland, MA, USA) and incubated for 24 h before imaging. To avoid possible edge effects from the glass bottom, the imaged cells were at least 100 μm above the glass bottom. Time-lapse images were collected every 5 min for 16 h using a Nikon TE2000 microscope equipped with *a* 10× objective (Nikon) and a Cascade 1K CCD camera (Roper Scientific, Tucson, AZ). Series of time-lapsed images were processed and analysed using the NIS-Elements AR software (Nikon) and a custom Matlab (Mathwork, Natick, MA) analysis software.

### Survival analysis

Using the Gene Expression-Based Outcome for Breast Cancer Online (GOBO) database (1881 breast cancer patient samples; http://co.bmc.lu.se/gobo) in which the gene expression were measured on Affymetrix HG-U133A arrays [50], we analyzed early-stage breast cancers in the early stage (165 breast cancer patient samples) where tumor grade is equal or smaller than 1and tumors are equal or smaller than 2.5 centimeters. The expression of individual laminin subunits (LAMA1, LAMA2, LAMA3, LAMA4, LAMA5, LAMB1, LAMB2, LAMB3, LAMB4, LAMB5, LAMC1, LAMC2, LAMC3) were totaled for each patient and then patients were stratified by high (in the top 25%) *vs*. low (in the bottom 25%) expression. Survival data was analyzed using a Kaplan Meier survival plot and *p*-values were calculated by log-rank testing.

### Real-time reverse transcription quantitative PCR (RT-qPCR)

RNA extraction and cDNA synthesis were performed as previously described [51]. The mRNA expression level of each integrin relative to 18S rRNA was calculated based on the threshold cycle (C_t_) as 2^−(ΔCt)^, where ΔC_t_ = C_t_ (target) − C_t_ (18S). Each experiment was performed three times in triplicate (*N* = 3 × 3). Gene expression values were normalized to ITGA1expression to show fold change in expression between different integrins. Primers used for the RT-PCR are displayed in supplementary Table S1.

### Statistical analysis

The number of cells examined for each experiment is indicated in the figure captions. Mean values ± s.e.m. and statistical analysis were analyzed using Graphpad Prism (Graphpad Software, San Diego, CA). Two-tailed unpaired Student's *t*-tests and one-way ANOVA were conducted to determine significance of samples with 2 groups and >2 groups, respectively as indicated by standard Michelin Guide (^****^P < 0.0001, ^***^P < 0.001, *P < 0.01, and *P < 0.05).

## SUPPLEMENTARY FIGURES, TABLE AND MOVIES


