# Supplementary material for: Normal mammary epithelial cells promote carcinoma basement membrane invasion by inducing microtubule-rich protrusions
Source: Oncotarget. 2015 Aug 3;6(32):32634–45. doi: 10.18632/oncotarget.4728 (PMC4741718; doi:10.18632/oncotarget.4728)
Supplement: Supplementary file 1 [file oncotarget-06-32634-s001.pdf]

SUPPLEMENTARY FIGURES, TABLE AND MOVIES

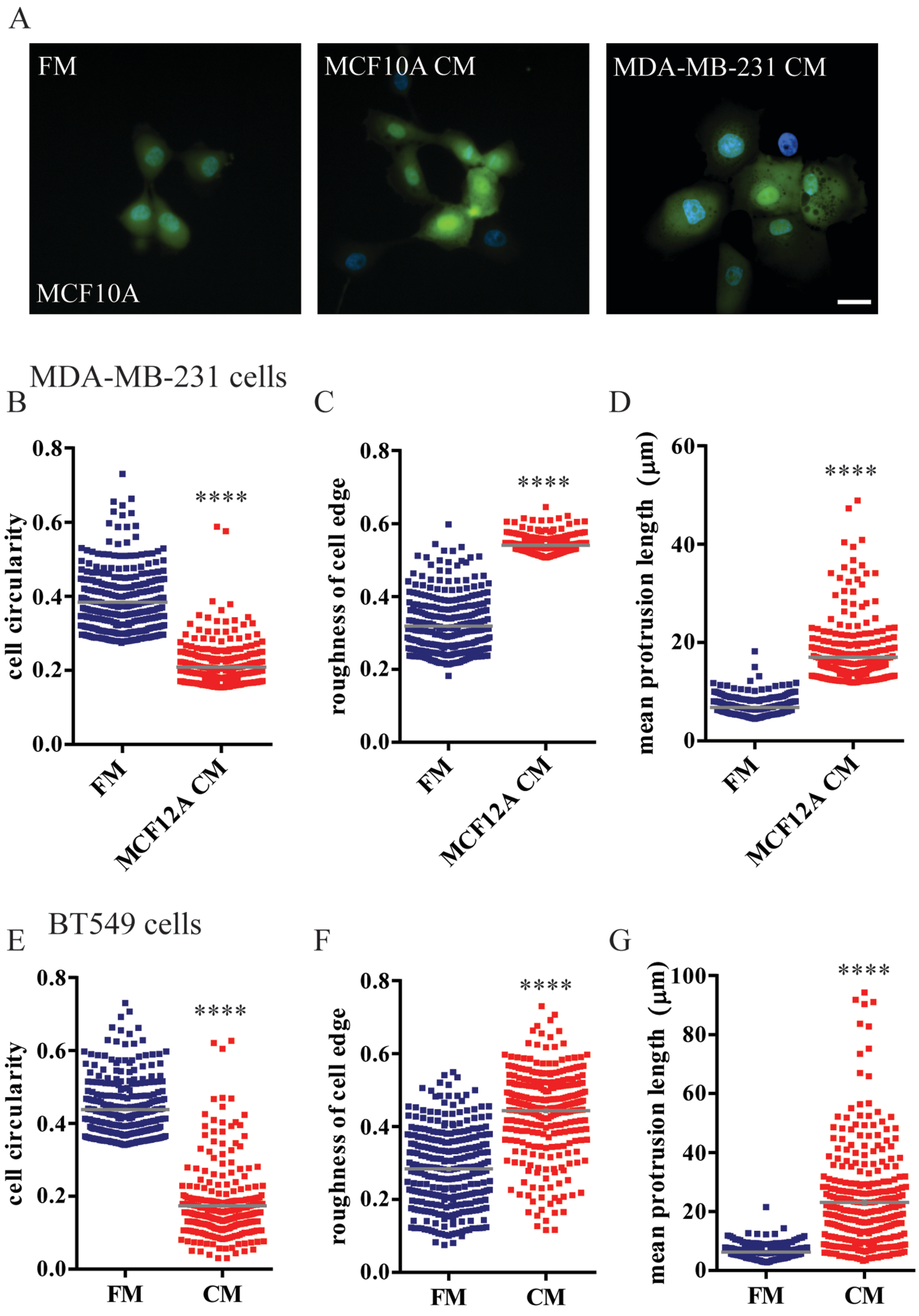

Supplementary Figure S1: Normal epithelium cells are insensitive to their own secreted proteins but switch breast carcinoma cells to a protrusive phenotype. A. MCF10A cells stably expressing EGFP were treated with regular fresh medium (FM) or conditioned medium (CM) harvested from either confluent MCF10A cells or confluent MDA-MB-231 cells. **B–D.** MDA-MB-231 cells were incubated with conditioned medium (CM) harvested from confluent human breast MCF12A epithelial cells. **E–G.** Breast BT549 carcinoma cells, isolated from primary tumors, were treated with conditioned medium (CM) harvested from confluent breast epithelial MCF10A cells. The dot plots represent (A and D) cell circularity, (B and E) cell roughness and (C, F) mean protrusion length. ****:$P < 0.0001$.

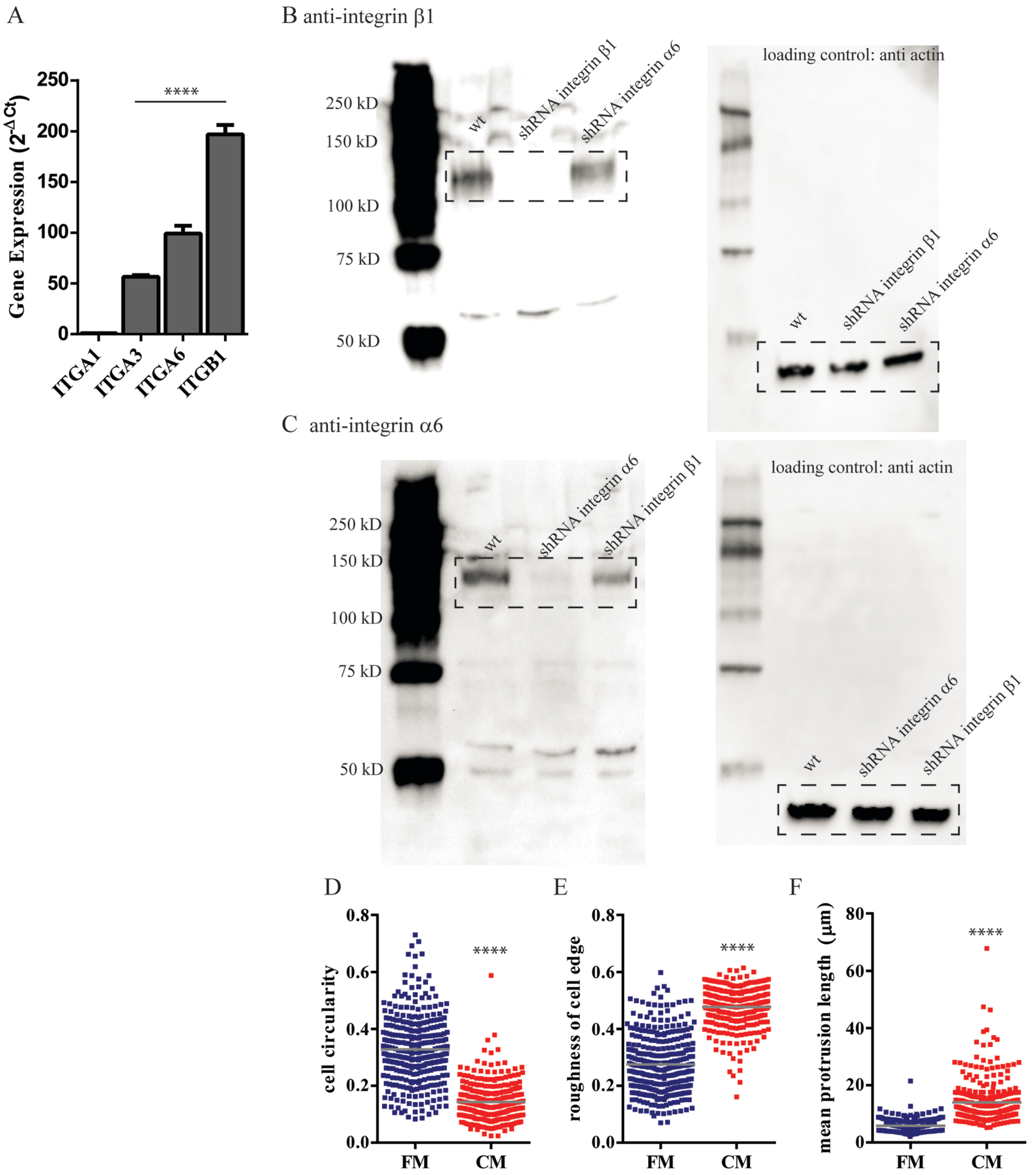

Supplementary Figure S2: The depletion of integrin α6 in carcinoma cells is not sufficient to inhibit the formation of protrusive phenotype induced by normal breast epithelial cells. induces a protrusive phenotype in breast carcinoma cells. A. Levels of *integrin* mRNAs were analyzed by RT-qPCR in MDA-MB-231 cells. **B–F.** MDA-MB-231 cells were transduced with recombinant lentivirus encoding for shRNA targeting *integrin α1 or β1* (B and C) The expression of integrin α6 and β1 were analyzed by western blotting using an antibody against integrin α6. The dot plots represent (D) circularity, (E) roughness of cell edges of cells and (F) mean length of control or α6 integrin-depleted MDA-MB-231 cells incubated with CM. ****:$P < 0.0001$.

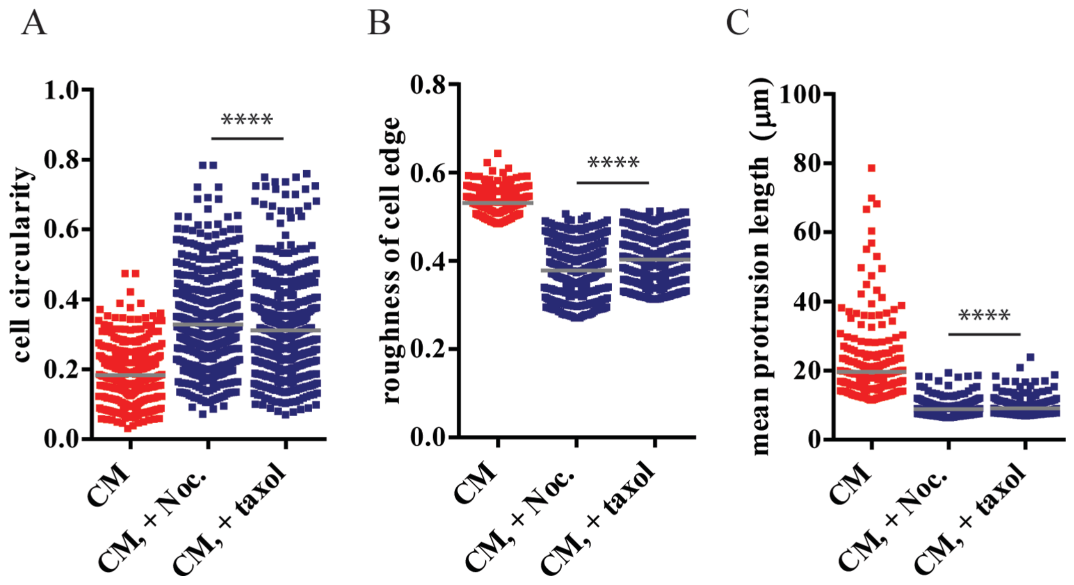

Supplementary Figure S3: Microtubule dynamics is required to maintain long protrusions in stimulated breast carcinoma cells. A–C. MDA-MB-231 cells were first incubated with MCF10A-CM for 16 h to initiate a protrusive phenotype. Stimulated cells were than treated with CM supplied with either the microtubule-depolymerizing drug nocodazole (125 nM) or the microtubule-stabilizing drug taxol (500 nM). The dot plots represent (A) cell circularity, (B) cell roughness and (C) mean protrusion length of BCCs. ****:$P < 0.0001$.

Supplementary Table S1: Primers used for the RT-PCR.

Gene		The sequence of primers
ITGA1	Forward	TTCAACCTCAATAAGTATTCTTCC
	Reverse	TCTCCATCTGTCACAATAACC
ITGA2	Forward	GGTCATCAGGGCACTATCC
	Reverse	CCAGAGTTGAACCACTTGTC
ITGA3	Forward	AAGGGACCTTCAGGTGCA
	Reverse	TGTAGCCGGTGATTTACCAT
ITGA5	Forward	CAGCCTTGCCAGAGATCCAA
	Reverse	TCCTTGTGTGGCATCTGTCC
ITGA6	Forward	TTGAATATACTGCTAACCCCG
	Reverse	TCGAAACTGAACTCTTGAGGATAG
ITGB1	Forward	ACTGATTGGCTGGAGGAATGTTAC
	Reverse	CTGGACAAGGTGAGCAATAGAAGG

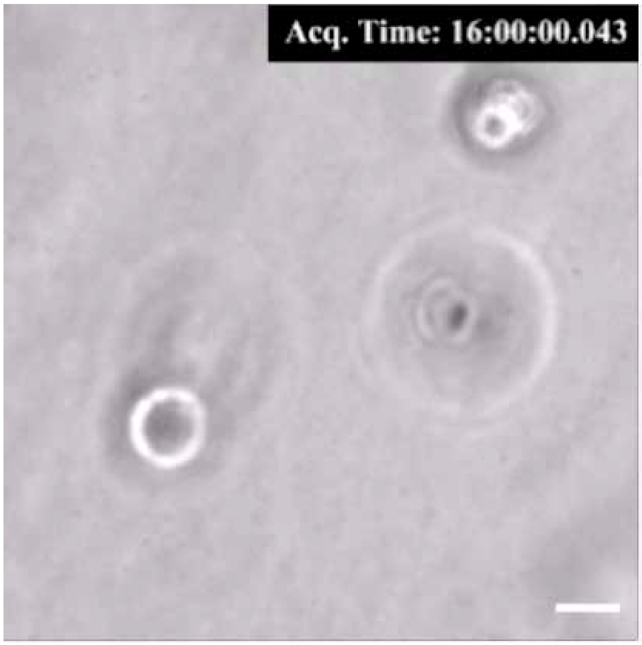

Supplementary Movie S1: The movement of MDA-MB-231 treated with regular fresh medium in a 3D matrigel. MDA-MB-231 cells were embedded inside three-dimensional (3D) matrigel in the presence of regular fresh medium (FM). The movie was made by collecting images every 5 min for 16 h. Scale bar, 20 µm.

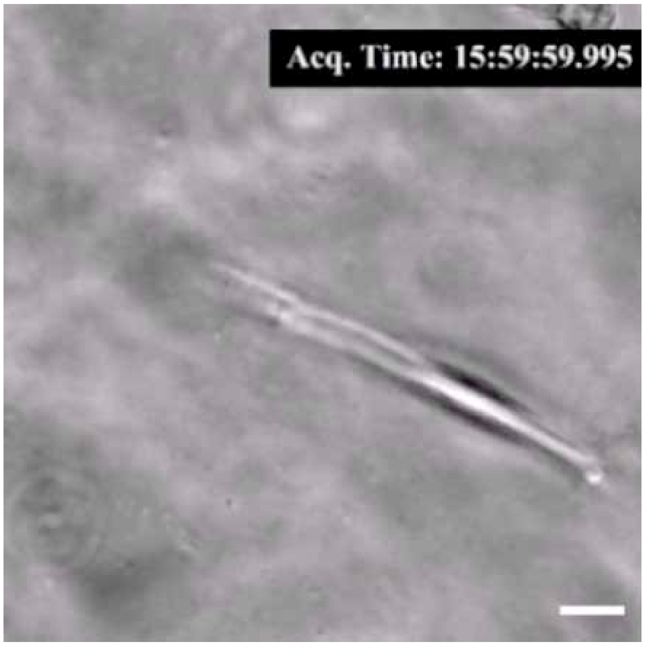

Supplementary Movie S2: The movement of MDA-MB-231 treated with MCF10A conditioned medium (CM) in a 3D matrigel. MDA-MB-231 cells were embedded inside three dimensional (3D) matrigel in the presence of MCF10A conditioned medium (CM). The movie was made by collecting images every 5 min for 16 h. Scale bar, 20 µm.

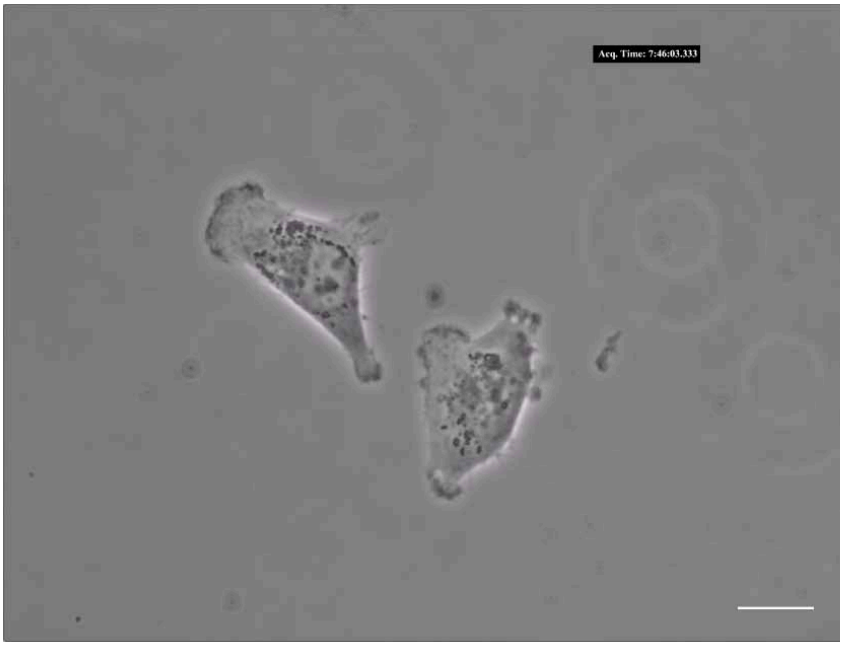

Supplementary Movie S3: The dynamic spreading morphology of MDA-MB-231 in regular fresh medium. MDA-MB-231 cells were seeded on the glass surface in the presence of regular fresh medium (FM). The movie was made by collecting images every 5 min for 16 h. Scale bar, 20 µm.

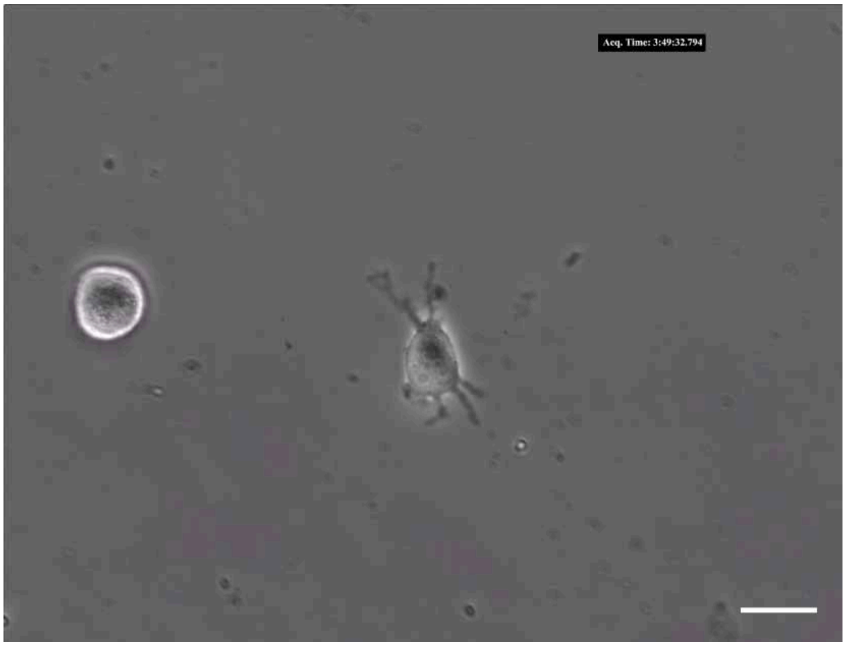

Supplementary Movie S4: The dynamic spreading morphology of MDA-MB-231 in MCF10A conditioned medium (CM). MDA-MB-231 cells were seeded on the glass surface in the presence of MCF10A conditioned medium (CM). The movie was made by collecting images every 5 min for 16 h. Scale bar, 20 µm.

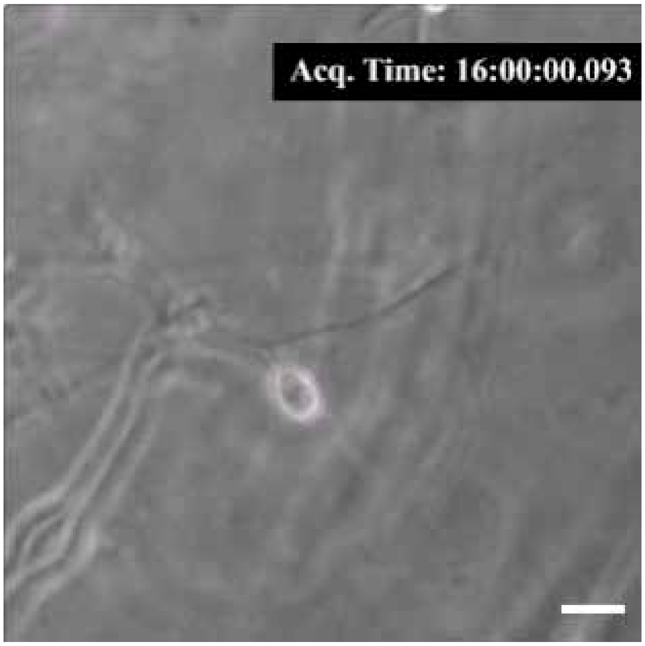

Supplementary Movie S5: The movement of MDA-MB-231 treated with CM supplemented with Y-27632 in a 3D matrigel. MDA-MB-231 cells were embedded inside three dimensional (3D) matrigel in the presence of MCF10A conditioned medium (CM) supplied with the ROCK inhibitor Y-27632. The movie was made by collecting images every 5 min for 16 h. Scale bar, 20 μm.
